# Relationship between plasma levels of sclerostin, calcium–phosphate disturbances, established markers of bone turnover, and inflammation in haemodialysis patients

**DOI:** 10.1007/s11255-018-2050-3

**Published:** 2018-12-24

**Authors:** Barbara Pietrzyk, Katarzyna Wyskida, Joanna Ficek, Aureliusz Kolonko, Rafał Ficek, Andrzej Więcek, Magdalena Olszanecka-Glinianowicz, Jerzy Chudek

**Affiliations:** 1 Pathophysiology Unit, Department of Pathophysiology, Medical University of Silesia, Katowice, Poland; 2 Health Promotion and Obesity Management Unit, Department of Pathophysiology, Medical University of Silesia, Katowice, Poland; 3 Department of Nephrology, Transplantation and Internal Medicine, Medical University of Silesia, Katowice, Poland; 4 Department of Internal Medicine and Oncological Chemotherapy, School of Medicine in Katowice, Medical University of Silesia in Katowice, 40-752 Katowice, Poland

**Keywords:** Chronic kidney disease-mineral and bone disorder (CKD-MBD), Sclerostin, Renal osteodystrophy (ROD), Calcium–phosphate disturbances, Haemodialysis patients

## Abstract

**Purpose:**

Data concerning the relation between increased levels of circulating sclerostin (a physiological inhibitor of bone formation) and bone turnover in patients with chronic renal failure (CRF) are limited. Therefore, the aim of this study was to evaluate associations between plasma sclerostin levels and calcium–phosphate disturbances, markers of bone turnover as well as inflammation in haemodialysis (HD) patients.

**Methods:**

In plasma samples obtained in 150 stable HD patients (92 men) aged 40–70 years, levels of sclerostin, fibroblast growth factor (cFGF23), osteocalcin, the N-terminal propeptide of type I procollagen, C-terminal telopeptide of the alpha chain of type I collagen (*β*-CTx), and inflammatory markers (IL-6 and TNF-α) in addition to routine parameters (calcium, phosphorus, parathyroid hormone—iPTH, 25-OH-D, alkaline phosphatase) were measured.

**Results:**

Plasma sclerostin concentrations were significantly higher in HD men than women (2.61 vs. 1.88 ng/mL, *p* < 0.01). Patients with sclerostin levels above median were characterized by lower iPTH and IL-6, but higher cFGF23 and TNF-α (significantly only in men) concentrations. Plasma sclerostin concentration positively correlated with serum 25-OH-D (*τ* = 0.204), phosphorus (*τ* = 0.1482), and TNF-α (*τ* = 0.183) and inversely with iPTH (*τ* = − 0.255), alkaline phosphatase (*τ* = − 0.203), IL-6 (*τ* =− 0.201), and *β*-CTx (*τ* = − 0.099) levels. In multivariate regression analysis, variability of sclerostin levels was explained by sex and 25-OH-D and phosphorus levels.

**Conclusions:**

Increased circulating sclerostin levels seem to reflect slower bone turnover in HD patients. Low levels of sclerostin are associated with vitamin D deficiency and good phosphates alignment.

## Introduction

Sclerostin is a protein secreted by osteocytes that prevents excessive bone formation [1]. It activates Wnt pathway that inhibits the differentiation of the mesodermal stem cells towards preosteoblasts and osteoblasts proliferation [2]. The sclerostin signal in osteoblasts is further modulated by several other factors, e.g. calcitriol, parathyroid hormone (PTH), glucocorticoids, and tumour necrosis factor-α (TNF-α). Calcitriol induces expression of low-density lipoprotein receptor-related protein 5 (LRP5) and inhibits bone marrow stromal cells, expression of Dickkopf-related protein 1 (DKK1) and secreted frizzled-related protein 2 (SFRP2)—antagonists of Wnt signalling pathway [3], and transcriptional regulation of key proteins osteoblasts, including non-collagenous proteins e.g. osteocalcin (OC) [4]. Simultaneously, calcitriol stimulates osteoclastogenesis by increasing the expression of receptor activator of nuclear factor kappa-B ligand (RANKL) and inhibiting the expression of osteoprotegerin (OPG) in preosteoblasts [5]. Previously, elevated sclerostin levels have been observed in subjects with a higher bone mineral mass [6]. It suggested that circulating sclerostin level is an important marker of the pool of mature osteocytes. As a consequence of greater bone mineral mass, circulating sclerostin levels are higher in men than in women and due to decreased clearance its level increases with the decline of renal function and age [7]. The sclerostin level is also considered as a potential biomarker of decreased bone formation.

In haemodialysis (HD) patients, bone biopsy remains a gold standard in the assessment of renal osteodystrophy (ROD). However, in daily clinical practice, several traditional biochemical surrogates of bone turnover are used. The assessment of iPTH, a bone isoenzyme of alkaline phosphatase (b-ALP), and 25-OH-D have an established position in the diagnosis of mineral and bone disorders in patients with chronic kidney disease (CKD-MBD) [8]. During the last decade, some novel potential markers of bone turnover, used in the diagnosis and management of osteoporosis, including osteocalcin, N-terminal propeptide of type I procollagen (P1NP)—markers of bone formation; C-terminal cross-linked alpha-chain telopeptide of type I collagen (CTx), N-terminal cross-linked telopeptide of the alpha chain of type I collagen (NTx), and pyridinoline and deoxypirydoline (PYD and DPD) [9] were extensively investigated, but to date, they are not widely applied in CKD patients.

Of note, in histomorphometric cross-sectional study performed in HD patients by Cejka et al., serum sclerostin concentration negatively correlated with parameters of bone turnover, as well as with osteoblastic number and function determined in bone biopsy [10]. Furthermore, an excessive accumulation of sclerostin was shown to inhibit PTH secretion in patients with chronic kidney disease [11], which may potentially slow an excessive bone turnover, induced by secondary hyperparathyroidism. On the other hand, sclerostin secretion by osteocytes seems to be also stimulated by the inflammation in HD patients [12].

The aim of this study was to evaluate the associations between plasma levels of sclerostin and calcium–phosphate metabolism disturbances, traditional and novel markers of bone turnover as well as inflammation in HD patients.

## Materials and methods

### Study population

For the purpose of this study, we performed additional tests in plasma samples from our previous project that enrolled 150 stable, selected HD patients (92 men and 58 women) with chronic renal failure (CRF), aged 40–70 years. The study protocol assumed a single withdrawal of an additional blood sample, while performing routine tests, before the midweek HD session on the morning after overnight fasting. Due to the requirement of morning blood samples collection, only patients on morning dialysis sessions were included. Plasma and serum samples were then stored at − 70 °C. Patients with previous history of gastrointestinal diseases, those receiving immunosuppressive medication, currently hospitalized and on HD therapy for less than 6 months were excluded as it was previously described [13]. The study protocol was accepted by the Bioethical Committee of the Medical University of Silesia in Katowice (KNW 22/KB1/185/I/11/12), and each patient gave informed consent for participation in the study. All patients were dialysed 3 times per week for 3.5 to 5 h (mean, 11.7 ± 0.9 h weekly). The structure of CRF causes was as follows: diabetic nephropathy (*N* = 42; 28.0%), glomerulonephritis (*N* = 24; 16.0%), hypertension (*N* = 17; 11.3%), interstitial nephritis (*N* = 13; 8.5%), autosomal dominant polycystic kidney disease (*N* = 10; 6.7%), nephrolithiasis (*N* = 8; 5.3%), other or unknown (*N* = 36; 24.0%). Patient characteristics including comorbidity, concomitant pharmacotherapy, and parameters of HD therapy are given in Table 1.

**Table 1:** Demographic and clinical characteristics of 150 haemodialysis patients (mean and 95% CI)

	All (*n* = 150)	Men (*n* = 92)	Women (*n* = 58)	Statistical significance men vs. women
Age (years)	62 (59–64)	62 (59–65)	61 (57–65)	NS
Body mass index (kg/m^2^)	26.1 (25.3–27.0)	26.6 (25.7–27.5)	25.4 (23.7–27.0)	NS
BMI ≥ 30 kg/m^2^ (*n*/%)	29/19.3	18/19.6	11/19.0	
Time on dialysis (months)	49 (41–56)	50 (39–61)	46 (36–56)	NS
Kt/V (per HD session)^a^	1.05 (1.01–1.08)	0.98 (0.94–1.02)	1.15 (1.09–1.20)	*p* < 0.001
Ultrafiltration (L)	2.5 (2.3–2.6)	2.5 (2.3–2.7)	2.5 (2.2–2.7)	NS
Primary cause of CKD (*n*/%)				
Diabetes	42/28.0	27/29.3	15/25.8	
Hypertension	17/11.3	13/14.1	4/6.9	
Nephrolithiasis	8/5.3	4/4.3	4/6.9	
ADPKD	10/6.7	4/4.3	6/10.3	
Ischemic nephropathy	2/1.3	1/1.0	1/1.9	
Glomerulonephritis	24/16.0	15/16.3	9/15.5	
Interstitial nephritis	13/8.5	3/3.2	10/17.2	
Other or unknown	34/22.7	25/27.5	9/15.5	
Co-morbidities (%)				
Hypertension	136/90.7	86/93.5	50/86.2	
Diabetes	55/36.7	36/39.1	19/32.6	
Coronary artery disease	83/55.3	57/62.0	26/44.8	
Stroke	12/8.0	9/9.8	3/5.2	
Past kidney transplantation	11/7.3	8/8.7	3/5.2	
CKD-MBD Pharmacotherapy (*n*/%)				
Oral phosphorous binders	143/94.7	89/96.7	54/93.1	
Carbonate calcium dose (g/day)	3.0 (2.6–3.5)	2.9 (2.4–3.5)	3.1 (2.4–3.8)	NS
Sevelamer hydrochloride	4/2.6	2/2.1	2/3.4	
Cinacalcet	17/11.3	8/8.7	9/15.5	
Cinacalcet dose (mg/day)	10.1 (5.1–15.1)	6.7 (1.6–11.9)	15.6 (5.3–25.8)	NS
Alfacalcidol	40/26.7	23/25.0	17/29.3	
Haemoglobin (g/dL)^a^	10.8 (10.6–11.0)	10.8 (10.6–11.1)	10.7 (10.4–11.0)	NS
Total cholesterol (mg/dL)	169 (160–177)	159 (148–170)	184 (171–198)	*p* < 0.01
LDL cholesterol (mg/dL)	90 (84–95)	84 (77–91)	98 (89–107)	0.01
HDL cholesterol (mg/dL)	28 (26–29)	27 (25–29)	28 (26–30)	NS
Triglycerides (mg/dl)	159 (142–177)	151 (127–175)	171 (147–196)	NS
Phosphorus (mmol/L)^a^	5.77 (5.52–6.02)	5.7 (5.4-6.0)	5.84 (5.4–6.3)	NS
Calcium (mg/dL)^a^	8.57 (8.44–8.70)	8.6 (8.4–8.7)	8.6 (8.3–8.8)	NS
Parathyroid hormone (pg/mL)^a^	446 (329–563)	376 (308–445)	565 (424–706)	*p* < 0.01
cFGF23 (RU/mL)^b^	1582 (1381–1782)	1615 (1352–1878)	1527 (1209–1846)	NS
25-OH-D (ng/mL)	12.8 (11.0–14.6)	14.8 (12.5–17.0)	9.6 (6.8–12.4)	*p* < 0.01
Osteocalcin (ng/mL)^b^	147 (133–160)	140 (124–156)	158 (133–182)	NS
total P1NP (ng/mL)^b^	312 (261–364)	271 (215–327)	379 (280–477)	*p* < 0.05
*β*-CTx (ng/mL) ^*^	1.65 (1.47–1.83)	1.61 (1.38–1.86)	1.69 (1.42–1.97)	NS
ALP (U/L) ^	115 (98–132)	109 (91–126)	124 (91–158)	NS
hs-CRP (mg/L)^b^	9.0 (7.3–10.8)	9.1 (7.0-11.2)	8.8 (5.8–11.9)	NS
IL-6 (pg/mL)^b^	6.7 (5.6–7.8)	7.5 (6.0–9.0)	5.37 (3.82–6.92)	*p* = 0.06
TNF-α (pg/mL)^b^	9.6 (7.6–11.6)	9.8 (7.6–12.1)	9.3 (5.3–13.2)	NS
Sclerostin (ng/mL)^b^	1.88 (1.23–3.01)	2.15 (1.44–3.25)	1.55 (0.99–2.47)	*p* < 0.01

*BMI* Body Mass Index, *ADPKD* Autosomal Dominant Polycystic Kidney Disease, *LDL* Low-Density Lipoprotein, *HDL* High-Density Lipoprotein, *cFGF23* C-terminal Fibroblast Growth Factor 23 Calcitriol, *1,25(OH)*_*2*_*D*_*3*_ 1,25-dihydroksycholekalcyferol, *P1NP* Procollagen I Aminoterminal propeptide, *β-CTx* C-terminal telopeptide, *ALP* Alkaline phosphatase, *hs-CRP* High-sensitivity C-reactive protein, *TNF-α* Tumour Necrosis Factor - α, *IL-6* Interleukin- 6

^a^Mean value from last 6 month

^b^Median (1Q-3Q)

### Laboratory measurements

In frozen plasma samples, sclerostin, cFGF23, 25-OH-D, osteocalcin, N-terminal propeptide of type I procollagen (total P1NP), and terminal C-terminal telopeptide of the alpha chain of type I collagen (*β*-CTx) were assessed. Commercially available ELISA kits were used for measurements of plasma levels of sclerostin (TECOmedical AG, Sissach, Switzerland; the mean intra- and inter-assay coefficients < 4.0% and the < 4.8%, respectively), cFGF23 (Immutopics. San Clemente, CA, U.S.; the mean intra- and inter-assay coefficients < 2.4% and < 4.7%, respectively), and 25-OH-Vitamin D (DRG Instruments GmbH for Hybrid XL, Marburg, Germany; the inter-assay precision < 14.2%). Osteocalcin, *β*-CTx, total P1NP, iPTH were assessed by ECLIA (Roche Diagnostics GmBH, Mannheim, Germany for Cobas e 411 analyser) with precision < 3.3%, < 4.2%, < 4.1%, and < 6.5%, respectively. The other parameters used in this analysis (calcium—Ca, phosphate—P, and alkaline phosphatase—ALP) were retrieved from medical records. Besides, some previously assessed inflammatory markers (IL-6 and TNF-α—R&D Systems, Minnesota, MN, U.S., hs-CRP—DRG GmbH, Marburg, Germany) were also included in the present analysis.

### Data analysis

The nutritional status was scored according to WHO criteria (obesity ≥ 30 kg/m^2^). Vitamin D status analysis was based on serum 25-OH-D concentration, and categorized as severe deficiency (< 10 ng/mL), deficiency (10–19.9 ng/mL), insufficiency (20–29.9 ng/ml), and sufficiency (≥ 30 ng/mL).

### Statistical analysis

Statistical analysis was performed with STATISTICA 11.0 PL StatSoft Corporation software (http://www.statsoft.com). The normality of quantitative variables distribution was checked by Shapiro–Wilk test. Results are given as mean values with standard deviation or 95% confidence intervals (95% CI) or medians with interquartile range (variables with skewed distribution). For comparison of groups, χ^2^ test (qualitative variables) and Kruskal–Wallis test were used, followed by Mann–Whitney *U* test (quantitative variables). The correlation coefficients were calculated according to Spearman. Multivariate regression analysis was performed for plasma sclerostin levels as dependent variable, with potentially explanatory variables selected on the basis of univariate analyses and including sex, serum phosphorus, 25-OH-D, TNF-α, iPTH, IL-6, and kT/V values. The value of *p* < 0.05 was considered as statistically significant in all analyses.

## Results

### Study population

Among 150 HD patients included in the study, the mean duration of dialysis treatment was approximately 4 years. Average BMI was 26 kg/m^2^, and 28 (19%) patients met the criteria for the diagnosis of obesity. Subjects diagnosed with diabetes consisted 36.7% of the study group. There were significantly higher total and LDL-cholesterol concentrations, as well as Kt/V per HD session values among women (Table 1).

### Correlates of plasma sclerostin

Median sclerostin levels were significantly higher in male than in female HD patients [2.15 (Q1-3: 1.44–3.25) vs 1.55 (0.99–2.47) ng/mL, *p* < 0.01; Table 1]. Diabetic male patients were characterized by significantly higher sclerostin levels [2.59 (1.86–3.33) vs. 1.90 (1.21–3.18), *p* = 0.04], whereas similar levels were shown in female patients [1.38 (0.95–2.61) vs. 1.56 (0.99–2.47), *p* = 0.79]. Biochemical data of male and female subgroups with sclerostin levels ≤ or > median values are shown in Table 2. Individuals with plasma sclerostin levels above median were characterized by lower concentrations of iPTH, IL-6, and serum HDL-cholesterol concentration in both gender subgroups, whereas plasma concentrations of cFGF23 and TNF-α were significantly higher, and haemoglobin level was lower only in men subgroup with plasma sclerostin concentration over median value (Table 2).

**Table 2:** Characteristics of men and women subgroups with plasma sclerostin levels ≤ median value and > median value (mean and 95% CI)

	Men (*n* = 92)		*p*	Women (*n* = 58)		*P*
	Sclerostin ≤ 2.15 ng/mL (*n* = 46)	Sclerostin > 2.15 ng/mL (*n* = 46)		Sclerostin ≤ 1.55 ng/mL (*n* = 29)	Sclerostin > 1.55 ng/mL (*n* = 29)	
Age	59 (54–63)	65 (61–68)	< 0.05	61 (55–68)	60 (54–66)	NS
BMI	26.7 (25.2–28.1)	26.5 (25.3–27.7)	NS	25.0 (22.9–27.2)	25.7 (23.0–28.4)	NS
Sclerostin (ng/mL)^b^	1.45 (1.17–1.83)	3.25 (2.72–4.60)	< 0.001	0.99 (0.84–1.22)	2.47 (1.86–3.05)	< 0.001
Haemoglobin (g/dL)^a^	11.2 (10.8–11.5)	10.5 (10.2–10.9)	< 0.05	10.8 (10.3–11.4)	10.6 (10.1–11.0)	NS
Total cholesterol (mg/dL)	157 (146–167)	162 (142–182)	NS	182 (165–200)	186 (165–207)	NS
LDL cholesterol (mg/dL)	84 (76–93)	84 (73–96)	NS	94 (83–106)	102 (87–117)	NS
HDL cholesterol (mg/dL)	30 (27–33)	25 (23–27)	< 0.01	30 (27–34)	26 (23–29)	0.06
Triglycerides (mg/dL)	135 (108–161)	168 (127–208)	NS	172 (132–212)	172 (142–202)	NS
Phosphorus (mmol/L)^a^	5.58 (5.11–6.05)	5.86 (5.47–6.25)	NS	5.65 (5.01–6.28)	6.05 (5.38–6.72)	NS
Calcium (mgld/L)^a^	8.52 (8.16–8.91)	8.60 (8.23–8.94)	NS	8.62 (8.25–9.0)	8.55 (8.21–8.89)	NS
Parathyroid hormone (pg/mL)^b^	209 (122–297)	125 (63–197)	< 0.01	248 (123–495)	116 (64–196)	< 0.01
cFGF23 (RU/mL)	1286 (920–1652)	1967 (1604–2331)	< 0.01	1369 (901–1836)	1692 (1240–2144)	NS
25-OH-D (ng/mL)	13.0 (9.85–16.15)	16.6 (13.3–19.8)	NS	9.4 (4.48–14.26)	9.8 (6.88–12.76)	NS
< 10 ng/mL (*n*/%)	23/50.0	16/34.8	NS	24/82.3	19/65.6	NS
10–19.9 ng/mL (*n*/%)	12/26.0	14/30.5		2/6.9	5/17.2	
20–29.9 ng/mL (*n*/%)	6/13.0	10/21.7		1/3.9	5/17.2	
≥ 30 ng/mL (*n*/%)	4/11.0	6/13.0		2/6.9	0/0	
Osteocalcin (ng/mL)^b^	138 (72–207)	118 (89–220)	NS	152 (74–191)	165 (72–260)	NS
total P1NP (ng/mL)^b^	174 (92–376)	177 (113–317)	NS	250 (127–542)	233 (99–412)	NS
*β*-CTx (ng/mL)^b^	1.48 (0.84–2.15)	1.26 (0.88–1.74)	NS	1.50 (1.07–2.30)	1.11 (0.67–2.40)	NS
ALP (U/L)	117 (93–142)	100 (73–126)	NS	99 (78–165)	99 (79–119)	NS
hs-CRP (mg/L)^b^	5.26 (3.05–10.3)	4.82 (2.58–16.44)	NS	5.00 (1.99–10.72)	3.72 (2.18–8.78)	NS
IL-6 (pg/mL)^b^	8.41 (5.45–11.1)	2.30 (0.68–10.45)	< 0.05	7.05 (5.34–8.77)	3.69 (1.14–6.23)	< 0.05
TNF-α (pg/mL)^b^	4.02 (2.13–8.36)	10.61 (5.71–16.80)	< 0.001	3.68 (2.36–10.1)	6.24 (3.85–9.08)	NS

*BMI* Body Mass Index, *ADPKD* Autosomal Dominant Polycystic Kidney Disease, *LDL* Low-Density Lipoprotein, *HDL* High-Density Lipoprotein, *cFGF23* C-terminal Fibroblast Growth Factor 23, *1,25(OH)*_*2*_*D*_*3*_ Calcitriol; 1,25-dihydroksycholekalcyferol, *P1NP* Procollagen I Aminoterminal propeptide, *β-CTx* C-terminal telopeptide, *ALP* Alkaline phosphatase, *hs-CRP* High-sensitivity C-reactive protein, *α TNF* Tumour Necrosis Factor, *IL-6* Interleukin- 6

^a^Mean value from last 6 months

^b^Median (1Q-3Q)

**Fig. 1:**
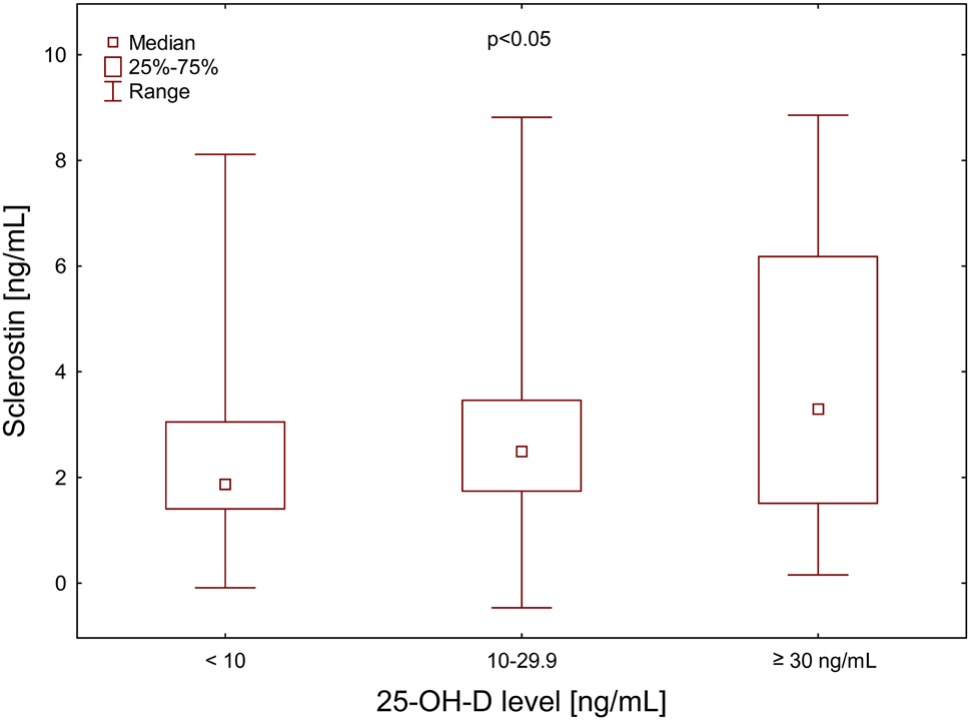
Plasma sclerostin concentration in relation to 25-OH-D ranges in haemodialysis men

Plasma sclerostin levels increased significantly along with 25-OH-D concentration intervals in men (Fig. 1, ANOVA < 0.05). There was also similar tendency among women: median plasma sclerostin concentration in 25-OH-D < 10 ng/mL subgroup was 1.65 (Q1-3: 0.96–2.23) and for 10–29.9 ng/mL subgroup it was 2.72 (1.56–3.36). However, 25-OH-D concentration ≥ 30 ng/mL was observed in 2 women, only.

**Fig. 2:**
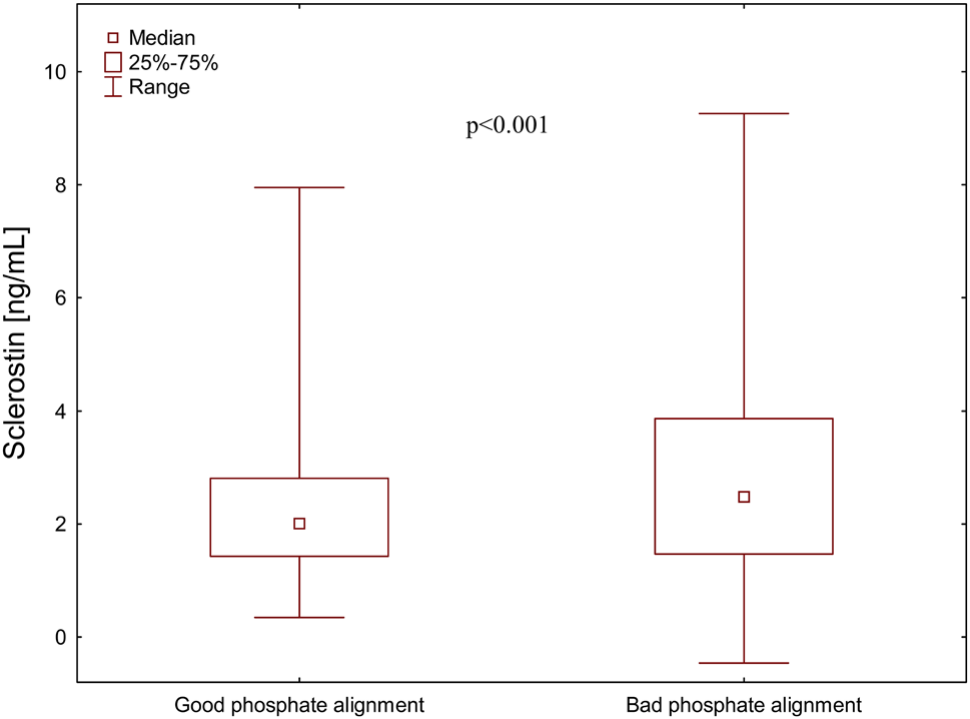
Plasma sclerostin concentration in relation to phosphate alignment in haemodialysis men

Median plasma sclerostin level was significantly higher in men with bad phosphate alignment (≥ 50% monthly phosphorus levels over 5 mg/dL) than in men with good phosphate alignment [2.48 Q1-3: 1.47–3.87, *n* = 62 vs. 2.01 (Q1-3: 1.43–2.81), *n* = 30 ng/mL; *p* < 0.001] (Fig. 2). There was no such difference in women 1.54 Q1-3: 1.07–2.43, *n* = 41 vs. 1.49 (Q1-3: 0.96–2.33, *n* = 16 ng/mL, respectively).

Plasma sclerostin levels positively correlated with serum phosphorus (*τ* = 0.148), 25-OH-D (*τ* = 0.204) and TNF-α (*τ* = 0.183), and inversely with iPTH (*τ* = − 0.255), *β*-CTx (*τ* = − 0.099), and IL-6 (*τ* = − 0.201) concentrations and serum ALP (*τ* = − 0.203) activity – Table 3.

**Table 3:** Selected correlations between sclerostin and inflammatory and bone formation markers

	Sclerostin	ALP	*β*-CTx	TP1NP	Osteocalcin
Age	*τ* = 0.069 *p* = 0.21	*τ* = 0.008; *p* = 0.89	*τ* = − **0.231**; *p* < 0.001	*τ* = − **0.288**; *p* < 0.001	*τ* = − **0.291**; *p* < 0.001
BMI	*τ* = 0.069 *p* = 0.22	*τ* = − 0.08; *p* = 0.20	*τ* = − **0.103**; *p* = 0.06	*τ* = − 0.037; *p* = 0.65	*τ* = − **0.162**; *p* = 0.05
kT/V	*τ* = − 0.101; *p* = 0.07	*τ* = 0.091; *p* = 0.12	*τ* = **0.096**; *p* = 0.08	*τ* = **0.219**; *p* = 0.007	*τ* = **0.258**; *p* = 0.001
Phosphorus	*τ* = **0.148**; *p* = 0.01	*τ* = − 0.018; *p* = 0.76	*τ* = **0.195**; *p* < 0.001	*τ* = **0.262**; *p* = 0.001	*τ* = **0.363**; *p* < 0.001
Calcium	*τ* = − 0.022; *p* = 0.70	*τ* = − 0.149; *p* = 0.01	*τ* = − 0.004; *p* = 0.94	*τ* = − 0.022; *p* = 0.79	*τ* = 0.044; *p* = 0.59
PTH	*τ* = − **0.255**; *p* < 0.001	*τ* = **0.154**; *p* = 0.009	*τ* = **0.402;***p* < 0.001	*τ* = **0.389**; *p* < 0.001	*τ* = **0.456**; *p* < 0.001
cFGF23	*τ* = **0.170**; *p* = 0.002	*τ* = − 0.073; *p* = 0.22	*τ* = **0.198**; *p* < 0.001	*τ* = **0.139**; *p* = 0.01	*τ* = **0.263**; *p* < 0.001
25-OH-D	*τ* = **0.204**; *p* < 0.001	*τ* = − **0.157**; *p* = 0.008	*τ* = 0.066; *p* = 0.23	*τ* = 0.009; *p* = 0.91	*τ* = **0.268**; *p* < 0.001
Osteocalcin	*τ* = − 0.004; *p* = 0.93	*τ* = **0.127**; *p* = 0.03	*τ* = **0.400**; *p* < 0.001	*τ* = **0.667**; *p* < 0.001	
TP1NP	*τ* = − 0.080; *p* = 0.15	*τ* = **0.278**; *p* < 0.001	*τ* = **0.428**; *p* < 0.001		*τ* = **0.667**; *p* < 0.001
*β*-CTx	*τ* = − **0.099**; *p* = 0.05	*τ* = **0.310**; *p* < 0.001		*τ* = **0.584**; *p* < 0.001	*τ* = **0.400**; *p* < 0.001
ALP	*τ* = − **0.203**; *p* < 0.001		*τ* = **0.310**; *p* < 0.001	*τ* = **0.278**; *p* < 0.001	*τ* = **0.127**; *p* = 0.03
Hs-CRP	*τ* = 0.007; *p* = 0.92	*τ* = 0.012; *p* = 0.83	*τ* = − 0.019; *p* = 0.73	*τ* = − 0.052; *p* = 0.53	*τ* = − 0.085; *p* = 0.30
Interleukin-6	*τ* = − **0.201**; *p* < 0.001	*τ* = 0.046; *p* = 0.43	*τ* = − 0.009; *p* = 0.87	*τ* = 0.064; *p* = 0.44	*τ* = − **0.147**; *p* = 0.07
TNF-α	*τ* = **0.183**; *p* < 0.001	*τ* = − **0.156**; *p* = 0.008	*τ* = − 0.036; *p* = 0.51	*τ* = − 0.009; *p* = 0.92	*τ* = **0.141**; *p* = 0.08

In multivariate regression analysis plasma sclerostin levels variability was explained by gender (*β* = 0.174; *p* < 0.05), 25-OH-D (*β* = 0.186; *p* < 0.05) and phosphorus levels (*β* = 0.180, *p* < 0.05).

## Discussion

Our study demonstrates a positive relationship between circulating sclerostin levels and both 25-OH-D and phosphates levels, whereas moderate negative correlations were present for only some markers of bone formation: serum ALP activity (a by-product of osteoblast activity), and resorption: *β*-CTx. Moreover, gender, 25-OH-D, and phosphorus level were shown to independently influence sclerostin level in HD patients.

In fact, CKD significantly affects plasma sclerostin level. It was shown that plasma sclerostin level increases with the progression of CKD stages, with the highest values in HD patients [14]. In addition, elevated sclerostin levels in the circulation were shown to decrease rapidly after successful kidney transplantation, which suggests that kidneys probably participate in sclerostin clearance [15]. However, it cannot be excluded that other mechanisms contribute to the increased sclerostin release by osteocytes in dialysis patients [15]. Among the potential factors, systemic inflammation, inevitable in HD patients, should be mentioned. Contrary to this hypothesis, our study shows a negative correlation between circulating sclerostin and IL-6 levels, that may suggest an inhibition of sclerostin production by the systemic inflammation. Surprisingly, we also noted an opposite, positive correlation between plasma sclerostin and serum TNF-α concentrations. It should be mentioned that IL-6 not only stimulates inflammatory processes, but also inhibits TNF-α production and its role in the inflammatory *milieu* is not fully elucidated. Nevertheless, such a contradictory association of TNFα and IL-6 with sclerostin levels may explain why in our study there was also no correlation between levels of sclerostin and CRP. Of note, Almroth et al. showed a positive correlation between serum sclerostin and TNF-α, but not CRP or IL-6 concentrations in a cohort of HD patients [12]. These conflicting data require further studies.

The relationship between plasma sclerostin levels and mineral and bone disorder (CKD-MBD) in HD patients has been studied previously. De Oliveira et al. showed an inverse association between serum sclerostin levels and the bone formation rate in 41 prevalent peritoneal dialysis patients [16], which was in line with *in vitro* experiments demonstrating inhibition of osteoblasts activity (reduction of serum ALP activity, synthesis of type I collagen and mineralization) by sclerostin [17]. Certainly, the circulating sclerostin levels are supposed to reflect the bone mass and changes in the bone microenvironment [18], however the direct evidence is missing. Generally, an inhibition of bone formation (osteogenesis) is usually followed by slowing of bone reabsorption. This phenomenon explains a negative correlation between plasma sclerostin levels in the circulation and markers of bone resorption, observed in our study (negative correlation between sclerostin and both ALP and *β*-CTx), and previously found in other clinical studies (negative correlation between sclerostin and *β*-CTx) [16, 19–22]. In addition, Behets et al. showed the highest levels of bone turnover markers in HD patients presenting the combination of high PTH with low sclerostin level [19]. The negative association between levels of sclerostin and iPTH concentrations, observed also in our study, may be rather explained by suppression of sclerostin production by osteocytes, or bone mass decline in secondary hyperparathyroidism. It should be mentioned that in animal models, PTH was shown to inhibit sclerostin expression in osteocytes [23, 24].

Regardless of pathophysiological aspects, the weak sclerostin correlations with traditional bone turnover markers revealed in our study (with correlation coefficients approx. 0.2) suggest that sclerostin measurement may not markedly improve the assessment of CKD-MBD in HD patients. The number of factors affecting the release of sclerostin and potentially its action on osteoblasts seems to limit its usefulness in the non-invasive models of bone metabolism assessment in HD patients.

One of potential confounders, revealed also by our study, is vitamin D status. It was shown that 1,25-OH-D increases the expression of sclerostin gene stimulating sclerostin synthesis and secretion [25]. In line with this finding, we showed the lowest plasma sclerostin levels in vitamin D deficient patients (with 25-OH-D levels < 10 ng/mL). However, data on the relation between vitamin D supplementation and sclerostin levels in CKD patients are inconsistent. Torino et al. showed that vitamin D receptor activation by paricalcitol causes a moderate increase in serum sclerostin in CKD G3-4 patients independently of age, gender and severity of renal dysfunction. This effect was lost after adjustment for iPTH, suggesting that it may serve to balance PTH suppression [26]. Inconsistently, Yadav et al. showed that high dose vitamin D supplementation did not affect serum sclerostin levels in non-diabetic stage G3-4 CKD patients [27]. Furthermore, Acibucu et al. demonstrated even a decrease in sclerostin level during intramuscular therapy with 300.000 IU cholecalciferol injected monthly for 3 consecutive months in vitamin D deficient non-CKD patients [28].

Of note, decline in renal excretory function is associated with accumulation of phosphorus and an increase in FGF23 level in the circulation [29]. It is unclear whether it is caused by increased production of FGF23 by osteocytes in response to accumulation of phosphate, or mainly decreased cleavage of the C-terminal FGF23 fragments [30]. Of interest, our data suggest that phosphorus also stimulates sclerostin production. This supposition is supported by the results of multivariate regression analysis that revealed male gender and phosphorus level as independent factors explaining plasma sclerostin level variability. The association between phosphorus and sclerostin has been already described in HD patients with low iPTH [31]. To our best knowledge, any phosphate-binding molecule on the surface of osteocytes/osteoblasts that regulates the production of sclerostin and FGF-23 has not been identified, yet.

The study has several limitations. The most important is the lack of bone mass assessment as well as bone biopsies. In addition, there was a low number of patients with optimal 25-OH-D levels, that potentially decrease statistical power of the association between sclerostin and vitamin D. A strength of our study is the assessment of wide range of markers that characterize bone turnover and calcium–phosphate metabolism and inclusion of inflammatory markers in the analysis.

In conclusion, increased circulating sclerostin levels seem to reflect slower bone turnover in HD patients. Low plasma levels of sclerostin are associated with vitamin D deficiency and good phosphates alignment in our cohort of haemodialysis patients. The number of factors affecting the release of sclerostin seems to limit its usefulness in the non-invasive assessment of bone metabolism in haemodialysis patients.
